# Analyzing Lung Disease Using Highly Effective Deep Learning Techniques

**DOI:** 10.3390/healthcare8020107

**Published:** 2020-04-23

**Authors:** Krit Sriporn, Cheng-Fa Tsai, Chia-En Tsai, Paohsi Wang

**Affiliations:** 1 Department of Tropical Agriculture and International Cooperation, National Pingtung University of Science and Technology, Pingtung 91201, Taiwan; krit22kmutt@gmail.com; 2 Department of Management Information Systems, National Pingtung University of Science and Technology, Pingtung 91201, Taiwan; 3 Department of Biochemistry and Molecular Biology, National Cheng Kung University, Tainan 70101, Taiwan; plusntsai@gmail.com; 4 Department of Food and Beverage Management, Cheng Shiu University, Kaohsiung 83347, Taiwan; 0627@gcloud.csu.edu.tw

**Keywords:** convolutional neural network, optimizer methods, lung disease, image classification, image processing, Mish activation function

## Abstract

Image processing technologies and computer-aided diagnosis are medical technologies used to support decision-making processes of radiologists and medical professionals who provide treatment for lung disease. These methods involve using chest X-ray images to diagnose and detect lung lesions, but sometimes there are abnormal cases that take some time to occur. This experiment used 5810 images for training and validation with the MobileNet, Densenet-121 and Resnet-50 models, which are popular networks used to classify the accuracy of images, and utilized a rotational technique to adjust the lung disease dataset to support learning with these convolutional neural network models. The results of the convolutional neural network model evaluation showed that Densenet-121, with a state-of-the-art Mish activation function and Nadam-optimized performance. All the rates for accuracy, recall, precision and F1 measures totaled 98.88%. We then used this model to test 10% of the total images from the non-dataset training and validation. The accuracy rate was 98.97% for the result which provided significant components for the development of a computer-aided diagnosis system to yield the best performance for the detection of lung lesions.

## 1. Introduction

The World Health Organization, using the latest statistics from the year 2018, reported that worldwide, there were 10.4 million patients and 1.6 million deaths from lung disease such as pulmonary tuberculosis. Lung disease is an infectious disease that causes a large number of deaths. Lung disease consists of many types such as pulmonary tuberculosis, pneumonia, effusion, mass, infiltration. lung disease often occurs in developing countries along with human immunodeficiency virus (HIV) and diabetes, which will immediately affect the immunity and infection of lung disease. This disease is a respiratory disease, meaning that it causes lung infections in the thoracic area of patients [1,2]. In early diagnosis by a doctor, chest X-ray (CRX) films are used. CRX films are used to determine the position and size of the lung disease in the chest. The patient’s lung examination uses CRX films, which almost all hospitals have available due to their inexpensive cost compared with magnetic resonance imaging (MRI) and computed tomography (CT) scans; thus, CRX films are a popular method for diagnosis as they can represent the organ structure inside the thoracic area in the body [3,4,5]. To summarize the results, thoracic CRX films are used to take images to diagnose the thoracic region. The patients infected by lung disease are numerous and still increasing in number and it takes a great deal of time for the doctor to diagnosis the disease due to a lack of radiologists. A computer-aided diagnosis (CAD) system is also used in lung disease screening [6].

Presently, doctors use CAD to reduce time for diagnosis disease and increase the convenience of diagnostics. A CAD system can be divided into three different basic technologies [7]. The first technology is image processing for extracting and enhancing the specific characteristics of the images, such as finding the lesions of the patient’s disease in the CXR films to learn and diagnose the location of the lung disease, as well as for training CAD schemes. CAD diagnostics are also inaccurate because the CRX films of each patient have different characteristics and anatomic structures, such as body fat or distorted bones. Various image processing techniques have been employed for various types of lesions. Some of the most commonly used techniques include filtering analysis according to morphologic filtering, Fourier transform, different image techniques, and transformations are also used and may cause a diagnosis fault [8].

The second technology is used to measure the quantitation of image features such as contrast, size and the shape of the lung lesions. It is possible to define many features as some mathematical formulas may not be easily understood by human observation. However, these formulas are generally helpful in determining, at least in the beginning phase of CAD development, the image features that are known and subjectively understood by radiologists. Further, the accuracy of their diagnosis is generally very high and reliable. One of the most important factors in the development of CAD schemes is to find the unique features that can distinguish between lesions and other normal anatomic structures [9,10].

The third type of technology is data processing of the differences between normal and abnormal patterns based on the image features. The simplest and most common method used in this step is a rule-based method, which may be established depending on the understanding of the lesion and other normal anatomic structures. Thus, it is important to understand that a rule-based method may provide useful information to improve CAD schemes. Other techniques used include discriminant analysis and decision-trees. It is our experience that a combination of a rule-based method with other methods like an artificial neural network (ANN) tends to produce the best results in terms of the high performance of the CAD system. As the basic concept of CAD is wide and general, CAD can be applied to all photographic styles, including conventional irradiation, CT, MRI, ultrasound imaging and nuclear medical imaging. CAD schemes, have been developed for many types of examinations, on all parts of the body, including the abdomen, chest, skull and the vascular system [11,12,13].

At present, many researchers improved the performance of the CAD system by using artificial intelligence (AI) technology, which is developing significantly, resulting in the technique used to analyze high-accuracy data. Deep learning uses the principle of machine learning, which can determine the functioning of the human brain. Data are used to create patterns for decision making, and deep learning can be applied to image processing analysis. Deep learning applies to various sciences, including medical diagnosis, and uses the convolutional neural network (CNN) model concept developed based on an ANN to select the features and classify the information that the CNN has developed to classify multimedia sources of data, such as video, sound, images and text, among others. Presently, the CNN’s structure adds greater performance, such as an activation function and an optimizer, which are significant components of the CNN’s structure. The traditional CNN model and various techniques have been developed to produce higher accuracy image classification and a greater speed of CNN processing for preprocessing, transfer learning, etc., as well as adjust the weights of image training from the ImageNet dataset and parameters in the CNN structure [14,15,16,17]. Moreover, transfer learning is important to give the CAD system the ability to accurately recognize images for diagnosis of the lung disease, that is, a dataset with enough data should be used for the training of a CNN model; the characteristics of the dataset must have features associated with the image’s recognition. Many organizations are aware of the importance of such a dataset for the large-scale training of up to 1.2 million or 1000 approximately image types to teach models more accurately to classify images, such as in ImageNet. Previous research also used transfer learning techniques to enhance high-accuracy models [18,19]. The development of the CNN model has increased its performance using transfer learning, but the CNN model is a complex construction. If the CNN model’s development uses incorrect patterns, the CNN model may reproduce false or inaccurate images. Computer processing is now faster than in previous periods and can support the CNN model with a more sophisticated structure, as well as large-scale dataset processing, though using transfer learning is still more popular [20].

## 2. Materials

### 2.1. Problem Definition

Lung disease is an epidemic that can be easily infected and cause many lung related problems in patients. X-ray method is popular treatment used to diagnose lung disease due to its efficiency and low price compared to other methods [5]. For example, many doctors and radiologists use an X-ray film to diagnose lung disease. As these are time taking, medical personnel are unable to respond for medical treatment on time [6]. Therefore, the use of CAD system can help medical screening to reduce the duty of medical personnel. Many researchers applied image analysis technology, such as Deep Learning with CNN models to improve the recognition of CAD system to analyze accurate images [9]. Deep Learning restrictions are inappropriate image feature that will be used to train CNN models, such as shape, size, color and dimension; moreover, the medical image is highly complex. Therefore, the diagnosis of CAD system is necessary to study the image analysis method efficiently by developing CNN models and image processing to be more efficient [14,15].

### 2.2. Dataset

**Figure 1:**
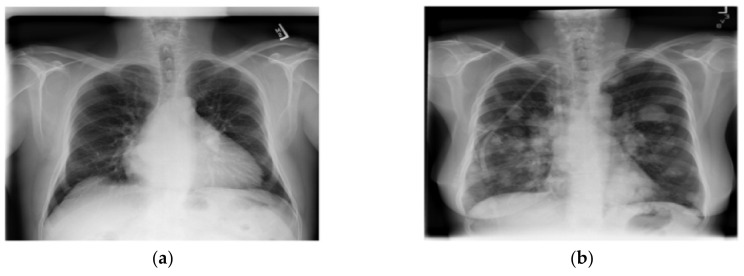
A normal lung (**a**) and a lung disease (**b**).

The dataset used in this research was taken from the National Institutes of Health Clinical Center-America’s Research Hospital [21], which contains X-ray images from patients. The X-ray image is two dimensions, black and white; and image size is different. The dataset divided into normal lung and a lung disease, which are open-access medical images. We selected this dataset that consists of 1000 normal lung images and 1500 lung disease images with different image features by adjusting the selected images with image processing method for appropriate image with training of CNN models in order to be able to learn the image features (color, size, dimension and shape) from X-ray images. The dataset contains images from the scoliosis patients’ spines are used for dataset training in the CNN models. An example of a normal lung and a lung disease is shown in Figure 1. 

### 2.3. Methodology

**Figure 2:**
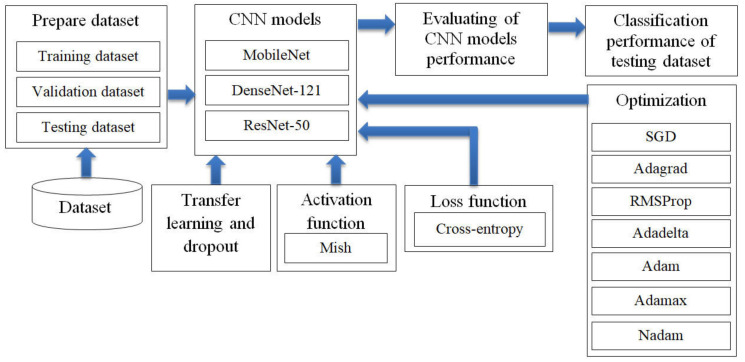
Research methodology: the optimization of convolutional neural network (CNN) models in predicting lung disease from chest X-ray images by using seven optimizer methods such as stochastic gradient descent (SGD).

The research divided this section into four parts. The first part involved preparation of the three CNN models for training and validating and testing the dataset. The second part involved transfer learning, dropout techniques and the Mish activation function to improve the efficiency of the CNN models. The third part used seven optimizer methods and Cross-entropy loss function to optimize the performance of CNN models in predicting lung disease from chest X-ray images. The fourth part involved evaluating CNN models performance for lung disease prediction from the chest X-ray film, as shown in Figure 2.

#### 2.3.1. Preparation of the CNN Models

##### MobileNet

**Figure 3:**
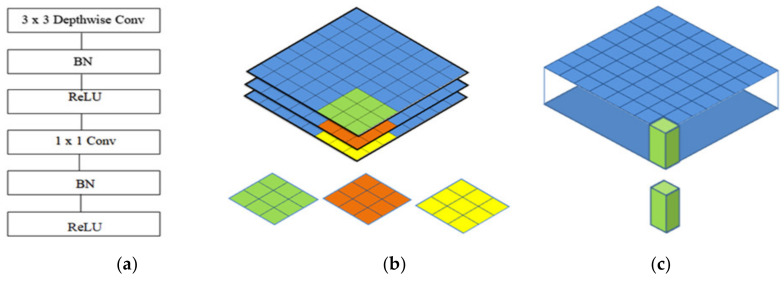
The architecture of MobileNet: (**a**) the convolutional layers of MobileNet; (**b**) the depth-wise convolution kernel; (**c**) the pointwise convolution kernel.

There are various deep learning architectures in the computer vision field. Many previous studies compared the accuracies of different architectures and determined the parameter values suitable to adjust the CNN architecture designed with computer vision. Computer vision emphasizes the accuracy and time savings, although it can also save the overuse of hardware. MobileNet architectures are added to convolutional layers with a kernel with size of 1 × 1 to decrease the number of times of multiplication iterations. Computer vision is determined by using a kernel with a size of 1 × 1 apply inception architectures within the MobileNet architecture. A new type of inception-based CNN architecture is called MobileNet. MobileNet uses inception architecture to build kernels of three dimensions with a size of 1 × 1 × M, called a depth wise convolution kernel; we build a pointwise convolution kernel, as shown in Figure 3a–c [22,23,24,25]. 

##### Resnet-50

**Figure 4:**
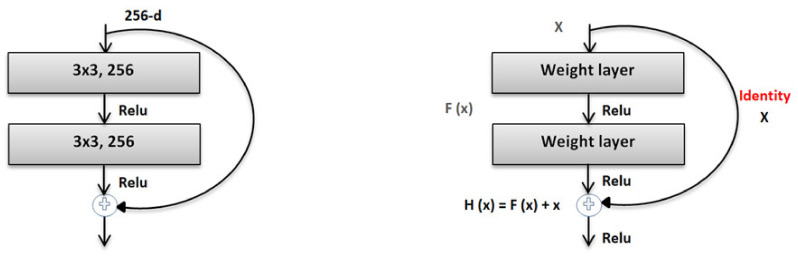
The architecture of ResNet-50.

The residual network (ResNet) was proposed by the Microsoft Corporation and won the 2015 ILSVRC competition. Global average pooling instead of fully connected layers is also used by ResNet-50, so the model of its 50-layer network is not too large. Convolution kernels of different sizes, such as 1 × 1 and 7 × 7, are used in the network to increase the diversity of its convolutions. Consequently, ResNet-50 has become very popular in the classification of image datasets. ResNet-50 elevates the concept of residuals where the image in the input goes directly to the output without experiencing a neural network. Consequently, the original image is maintained. This shortcut connection of ResNet-50 was introduced via the basic idea of a deep residual network, which can pass over some layers. In addition, two mappings were proposed in ResNet-50, one is identity mapping, and the other is residual mapping [26,27], as shown in Figure 4. 

##### DenseNet-121

**Figure 5:**
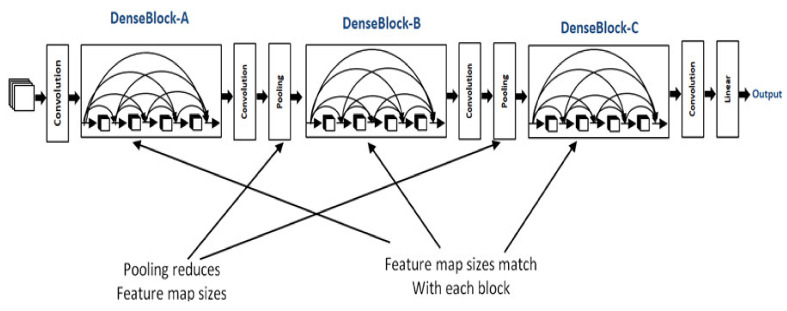
The architecture of DenseNet-121.

In DenseBlock, each layer has feature maps of an equal size, and within the channel dimension, it is possible for these DenseBlocks to be connected. The function for a nonlinear DenseBlock employs a BN + ReLU + 3 × 3 convolution. It is also important to consider the different types of ResNet; after all, DenseBlock convolutions of the individual layers, the output characteristics of the images are the image features. Assuming a number of channel input feature map layers, the feature is then entered into the channel layer, which increases the number channels of layers. Thus, despite setting a smaller input, DenseBlock is large as a consequence of the features resulting from reuse, whereby each layer has own unique features, this is known as a DenseNet-A structure. The latter layer will have a rather large input and therefore, the interior portion of the DenseBlock can make use of an additional layer that acts as a bottleneck in order to reduce the number of arithmetical manipulations required, primarily through the addition of a 1 × 1 convolution to the initial form, as can be seen in Figure 5, specifically for the BN + ReLU + 1 × 1 Conv + BN + ReLU + 3 × 3 convolution, which is known as a DenseNet-B structure. When a 1 × 1 convolution-obtained image feature seeks to lower the number of features, the computational efficiency is improved [28,29,30], as shown in Figure 5.

#### 2.3.2. Improvement of CNN Models Efficiency

##### Transfer Learning

**Figure 6:**
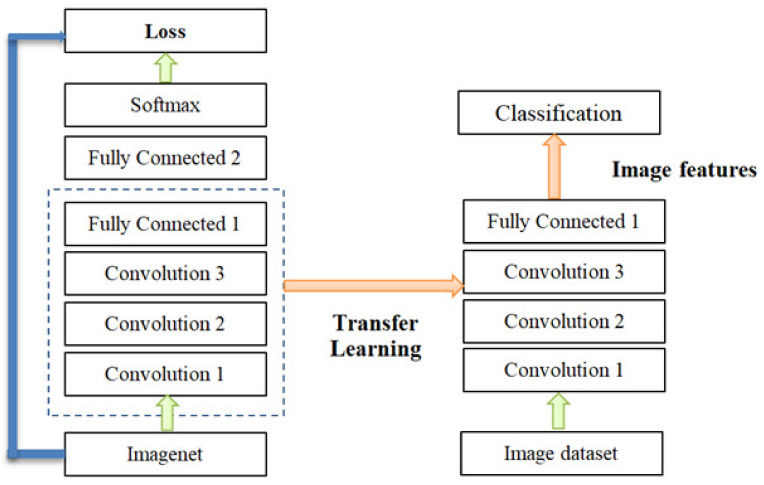
Convolutional neural network (CNN) transfer learning.

During its training and validation, a CNN model has a complex structure, which involves many Parameters (Weights); thus, the CNN model has a complex structure. For the initial training and validation of transfer learning, we used a large dataset (the ImageNet dataset), containing 1000 classes or 1.2 million images, which is effective for timely and accurate image classification [19,20]. This research takes advantage of transfer learning to improve the CNN models as shown in Figure 6.

##### Dropout Technique

**Figure 7:**
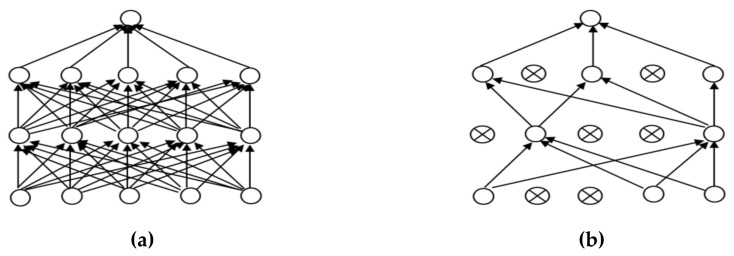
Dropout: (**a**) a neuron before a dropout; (**b**) a neuron after a dropout.

In order to make the CNN model more effective for the classification of images we adjusted the parameter values to suit the dataset for the training model; these parameters included batch size, activation function and the optimizer. Another main problem of CNN models with complex structures is overfitting. In this research, the dropout technique is used to solve the overfitting problem. Teaching a neuron network requires a large dataset, which is used to train the systems several times to create a deep model for classifying accurate images. However, there is the possible risk of overfitting, which may cause the model to classify inefficient images [31,32]. However, a dropout can prevent overfitting, as shown in Figure 7. 

##### Mish Activation Function

Mish is superior to ReLU at high significance levels (*p* < 0.0001). Mish has been tested using more than 70 benchmarks, including image classification, segmentation and generation and has been compared with 15 other activation functions. In addition, the Mish function also guarantees the smoothness of each point. The characteristics of the self-selection gate are able to replace activation functions such as ReLU (point function), as shown in Formula (1) [33]. These functions can receive a single scalar to change the network parameters without entering any scalar. Mish takes inspiration from swish’s self-gating property, where scalar input is provided to the gate. Self-gating is able to replace activation functions like ReLU without changing the network parameters. Variable (x) in Formulas (1) and (2) represents the input values of the activation function. Mish has no upper bound but does have a lower bound; Mish also has smooth and non-monotonic properties that improve the results [34], as shown in Formula (2).

(1) $$\mathrm{ReLU}\left(x\right)=max(0,x)$$

(2) $$\mathrm{Mish}\left(x\right)=x\times tanh\left(softplus\left(x\right)\right)\;$$

#### 2.3.3. Loss Function

The function of the loss function layer (loss layer) is to calculate the expected results predicted by the key features, to make differential corrections with the real results, and to combine the gradient drop optimization function to increase the convergence speed of network weight renewal. The most commonly used correction functions are loss functions, such as mean absolute error (MAE), mean square error (MSE) and cross-entropy [35]. This experiment used (cross-entropy) as shown in Formula (3).

(3) $$f\left(x;u\right)=exp\left(-\sum_{j=1}^{N}\frac{xj}{uj}\right)\prod_{j=1}^{N}\frac{1}{uj}$$

Cross-entropy was used to determine the sample to be calculated, which consisted of *x_j_* with *u_j_* values, where *x_j_* represents random weights, and *u_j_* represents the weights, which are exponentially distributed and independent of each other.

#### 2.3.4. Evaluating CNN Model Performance for Lung Disease Prediction from the Chest X-ray Film.

This study utilized several common parameters to evaluate architectural deep learning examination performance using three convolutional neural networks. TP indicates true positive (True Positive) (i.e., predicted to suffer from lung disease and actually suffering from lung disease), while TN is true negative as predicted to suffer from lung disease and actually suffering from lung disease, while TN is true negative (True Negative) (i.e., the predicted absence of lung disease and no recorded presence of lung disease). FP is a false positive, which predicts the development of lung disease that is not actually present, while FN is a false negative which predicts no development of lung disease despite the real presence of lung disease, the Formulas (4)–(7) based on the work in [36,37,38,39].

1. Accuracy can be represented as the number of classified data sets divided by the total number of data test sets, as shown in Formula (4).

(4) $$\mathrm{Accuracy}=\frac{TP+TN}{TP+TN+FP+FN}$$

2. The precision rate indicates the correct prediction of the number of categories divided by the total number of data falling into that category, as shown in Formula (5).

(5) $$\mathrm{Precision}=\frac{TP}{TP+FP}$$

3. The recall rate (Recall) correctly predicts the number of categories divided by the total number of data actually belonging to each category, as shown in Formula (6).

(6) $$\mathrm{Recall}=\frac{TP}{TP+FN}$$

4. The F1measure is used to balance the assessment of accuracy and recall rates and also to evaluate classification models, as shown in Formula (7).

(7) $$F1measure=\frac{2TP}{2TP+FP+FN}$$

## 3. Implementation Details

### 3.1. Computer Hardware and Software Setting.

In this experiment we created CNN models using computer hardware and software as the execution environment, as shown in Table 1 below.

### 3.2. Dataset Setting

Due to the chest X-ray film using a wide range of sizes and a large size not being suitable for dataset training to create the CNN models, it is necessary to shrink the X-ray image to reduce time for training the CNN models. This process reproduces the model and converts the image to a matrix size of 224 × 224 × 3, which is the normal size for dataset training in CNNs. Although the X-ray image is black and white, the data are red, green, and blue using an RGB three-color system [40]. 

**Figure 8:**
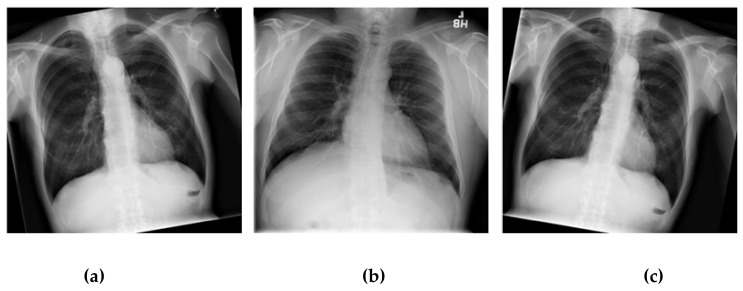
Examples of distorted chest X-ray images: (**a**) the patient’s normal spine tilted −10 degree by rotating; (**b**) a patient’s abnormal spine; (**c**) a patient’s normal spine tilted 10 degree by rotating.

The technique of adding the number of the images in a dataset via the data augmentation technique is a long-standing technique that solves datasets of a small number. There are several lung shapes other than a normal lung. In fact, the chest X-ray image has a slightly distorted angle from the original images, though not over 90 degrees. This model cannot be applied effectively considering the actual performance of the original dataset [41,42]. This research has applied the data augmentation technique, which will rotate only some of the images. In order to apply the augmentation technique to the chest X-ray dataset and not to distort the original image for improving the dataset efficiency to use training the CNN models, we used the shuffle sampling technique in combination with the rotated images. The angle will be random for each image in the range. The duplicate images of the dataset reduce this problem. Using only the shuffle sampling technique reduces the duplicate images in the dataset [43,44]. Sometimes an error occurs, such as an image being taken with a tilted angle, the presence of a distorted lung shape in a normal image, or a patient with a scoliosis spine. In order to develop a modeling experiment that can manage an image better without having to take a tilted image, the scoliosis patients’ spines are used for dataset training in the model. Due to the distorted X-ray image not being present in the dataset used in this research, it is possible to create a chest X-ray image that represents a distorted X-ray image by rotating it. The image is assigned an angle of −10 to 10 degrees randomly based on the dataset training [45,46]. Figure 8 illustrates comparative images of the lung shapes of the images created with the patient’s scoliosis spines. Some parts of the lungs have similar shapes. The images on the left and right are the chest X-ray images generated by image rotation, while the middle side is the chest X-ray image of the patient with an abnormal spine [47,48].

### 3.3. Optimizer Setting

The gradient descent method is presently the most famous optimizer method and is also the most commonly used method for optimizing a CNN. The latest machine learning libraries contain various algorithms for enhancing the gradient descent method, but these algorithms are not disclosed and are used as black box optimizers to develop the performance of a CNN. This experimental research used seven famous optimizer methods, the formulas of which are shown below.

1. Stochastic Gradient Descent (SGD) updates a high variance value impact into a loss function value with direct variation and different intensities. This is a good method because it easily and efficiently obtains the minimum value in the center of the field compared to the other algorithms [29]; the formula is shown below.

(8) $$\theta =\theta -\eta \cdot \nabla_{\theta }J\left(\theta ;\chi^{i};y^{i}\right)$$

For SGD, the determined learning rate is 0.1 (*η*), the input is *ꭓ^i^*, and the label is *y^i^* for the training, the gradient for loss function uses *_θ_J*, and the validation dataset. *θ* is the cost function of the calculating gradient.

2. Adagrad is an algorithm that can optimize the learning rate for the parameter in a suitable range by increasing its updating for a smaller number of parameter values. However, little time is used to update the various numbers of the parameter [30]; the formula is shown below.

(9) $$\theta t,i=\theta t-1,i-\frac{\eta }{\sqrt{Gt-1,i+\varepsilon }}.gt-1,i$$

Adagrad’s learning rate is 0.1 (*η*). *G_t_*_-1, i_ is the gradient of the objective function (*θ_t_*) for the calculating gradient at time step *t*, *ε* = *le^-^*^08^ and *g_t_* is the current gradient.

3. Adadelta can constrain the collection of the calculation of gradient descent to resize the resulting weight value instead of collecting the W value from the previous update. The aim is to repeat the decaying learning rate of all previous gradients [49]; the formula is shown below.

$$E\left[g^{2}\right]t=\gamma E\left[g^{2}\right]t-1+\left(1-\gamma \right)g^{2}t$$

The Adadelta learning rate is 0.1 (*η*); the solving fraction problem (*rho*, or *γ*) of the gradient is 0.9 at time step *t*, the diagonal matrix is *g*^2^*t*, and the decaying average is *E*[*g*^2^]*t*. 

4. RMSprop is a method for collecting the cost value of the gradient descent that is used for learning by applying the *gt* rate, MeanSquaret and *x* represent the historical learning rate and solves the problem of Adagrad’s radical reduction in learning rates [32]. The formula is shown below.

$$E\left[g^{2}\right]t=0.9E-\left[g^{2}\right]t-1+0.1g^{2}t$$

The RMSprop learning rate is 0.001 (*η*). We used Hinton’s input to set the solving fraction (*rho* or *γ*) of the gradient as 0.9 at time step *t*, the diagonal matrix is *g*^2^*t* and the decaying average was set as *E*[*g*^2^]*t*. 

5. Adaptive Moment Estimation (Adam) is an optimizer that can adjust the learning rates for each parameter at a time. It can also solve the decay of the gradients in each subsequent step along with Adadelta and explain the origination of decaying m_t_, as well as gradients [50]. The formula is shown below.

(10) $$m_{t}=\beta_{1}m_{t-1}+\left(1-\beta_{1}\right)gt$$.

To create the vectors of Adam using *m_t_* at time step *t*, we set *β*_1_=0.9 according to the advice provided by the authors of Adam.

6. Adamax is a variant of Adam and provides a simpler range for the upper limit of the learning rate. This model reduces the unstable problems of the parameter values. The formula is shown below.

(11) $$v_{t}=max\left(\beta_{2}\times vt-1,\left|gt\right|\right)$$

The Adamax learning rate is 0.002 (*η*), based on the work in [50] using *β*_2_ = 0.999, where |*gt*| is the current gradient and *v_t_* is the update rule scales of the gradient in Adamax.

7. Nadam is similar to Adam with Nesterov momentum. It has a stronger constraint in its learning rate and also has a more direct impact on the update of the gradient. The formula is shown below;

(12) $$\theta t+1=\theta t-\frac{\eta }{\sqrt{\overset{\wedge }{u_{t}}}+\varepsilon }\left(\beta_{1}{\overset{\wedge }{a}}_{t}+\frac{\left(1-\beta_{1}\right)gt}{1-\beta_{1}^{t}}\right)$$

The Nadam learning rate is 0.002 (*η*), based on the work in [51] using *β*_1_ = 0.9, *ε* = *le^-^*^08^ and the objective function ($\theta_{t}$), with *â_t_* and *û_t_*, provide an updated rule for Nadam at time step t. 

### 3.4. Parameter Setting for Training and Validation

This experiment uses three famous CNN models, with input size of 224 × 224 and seven optimizer methods for optimization of these CNN models. The iterations are 70 epochs, the training dataset size is 80% and the validation dataset size is 20% of the total number of the lung disease dataset; the convolutional neural network output layer contains two classes that comprise the normal and lung disease status. The batch size is 20 images, the activation function is Mish, the loss function is Cross-entropy, the dropout technique for solving the overfitting problem is 0.5, based on [31,32]. Table 2 shows a list of the learning rates of each optimizer method. The parameter values of the learning rates based on the work in [49,50,51,52,53].

## 4. Experimental Results

Table 3, Table 4 and Table 5 illustrate the performance of the CNN models combined with the seven optimizers and Mish comparison with traditional CNN models. Table 3 shows the lung lesion detection performance of MobileNet with Mish compared to traditional MobileNet, which uses ReLU. The best results with MobileNet were obtained by using Nadam and Mish, with an accuracy rate of 93.28%, a precision rate is 93.24%, a recall rate of 93.46% and an F1 measure rate of 93.27%. For MobileNet using SGD with ReLU (traditional method), the accuracy rate was 74.48%, the precision rate was 75.93%, the recall rate was 73.48%, and the F1 measure rate was 73.52%.

**Figure 9:**
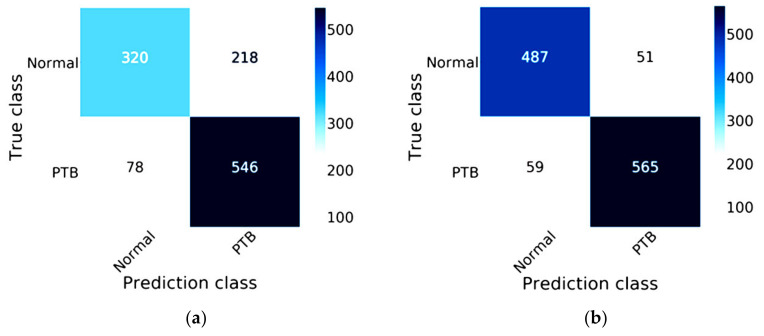
Confusion matrix of MobileNet: (**a**) the traditional model results of MobileNet; (**b**) the results of MobileNet combined with Mish and Nadam.

Figure 9 compares the efficiency between MobileNet and MobileNet combined with Mish and Nadam on the validation data, which comprises 20% of the 5810 images, or 1162 images. These images are split into two statuses, 538 images of a normal status and 624 images of a lung disease status for predicting lung lesions. The true class of confusion matrix for MobileNet combined with Mish and Nadam is shown in Figure 9b, which correctly predicted a normal status in 487 images and a lung disease status in 565 images; this model did not correctly predict lung lesions in 110 images. The MobileNet results are shown in Figure 9a, this model correctly predicted a normal status in 320 images and a lung disease status in 546 images. 

For ResNet-50, the best results were obtained by using Nadam and Mish, with an accuracy rate of 97.59%, a precision rate of 97.52%, a recall rate of 97.74%, and an F1 measure rate of 97.58%. For ResNet-50 using SGD with ReLU (the traditional method), the accuracy rate was 79.43%, the precision rate was 79.34%, the recall rate was 79.24%, and the F1 measure rate was 79.28%, as shown in Table 4.

**Figure 10:**
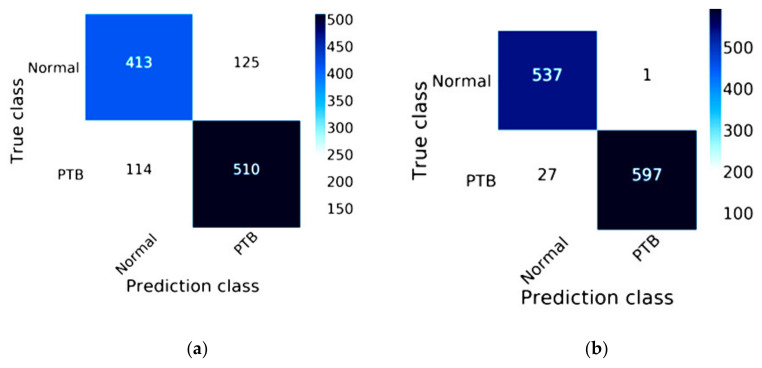
Confusion matrix of ResNet-50: (**a**) the traditional model results of ResNet-50; (**b**) the results of ResNet-50 combined with Mish and Nadam.

The efficiency of ResNet-50 combined with Mish and Nadam is shown in Figure 10. The true class of the confusion matrix for ResNet-50 combined with Mish and Nadam is shown in Figure 10b. This model correctly predicted a normal status in 537 images and a lung disease status in 597 images of lung disease status and did not correctly predict lung lesions in 28 images. ResNet-50 is shown in Figure 10a; this model correctly predicted a normal status in 413 images and a lung disease status in 510 images.

**Figure 11:**
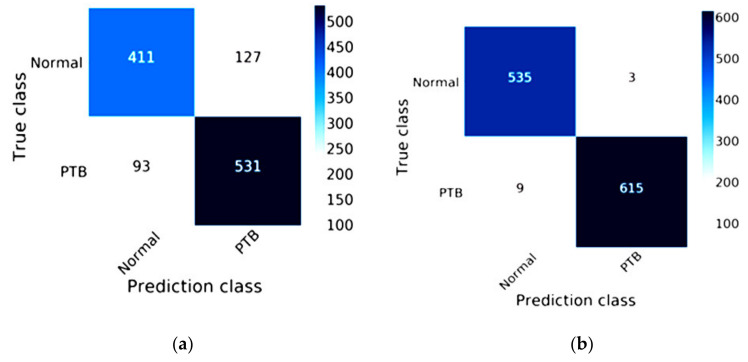
Confusion matrix of DenseNet-121: (**a**) the traditional method results of DenseNet-121; (**b**) the results of DenseNet-121combined with Mish and Nadam.

The efficiency of DenseNet-121 combined with Mish and Nadam is shown in Figure 11. The true class of the confusion matrix for DenseNet-121combined with Mish and Nadam is shown in Figure 11b. This model correctly predicted a normal status in 535 images and a lung disease status in 615 images and did not correctly predict lung lesions in 12 images. The DenseNet-121 results are shown in Figure 11a; this model correctly predicted a normal status in 411 images and a lung disease status in 531 images.

Table 5 shows the best performance for the detection of lung lesions using the optimizer method with the activation function, which can increase the potential of CNN models. In this research, using Nadam and Mish combined with DenseNet-121 predicted lung lesions with an accuracy rate of 98.88%. The precision rate was 98.83%, the recall rate was 98.91%, and the F1 measure rate was 98.87%. This is a higher accuracy rate than the prediction of lung lesions with the traditional method of DenseNet-121 which uses ReLU with SGD and offers an accuracy rate of 81.06%, a precision rate of 81.12%, a recall rate of 80.74%, and an F1measure rate of 80.86%, as shown below.

Table 6 describes the 580 testing images of the training and validation data, split into 232 images of a normal status and 348 images of a lung disease status from chest X-ray images. The traditional method of DenseNet-121 produced false predictions for 8 images of a normal status or 3.44% and 8 images of a lung disease status or 2.30%; the true predictions included 224 images of a normal status or 96.56% and 340 images of a lung disease status or 97.70%. DenseNet-121 combined with Mish with Nadam produced false predictions for 3 images of a normal status or 1.29% and 3 images of a lung disease status or 0.87%; true predictions were made for 229 images of a normal status or 98.71% and 345 images of a lung disease status or 99.13%.

## 5. Discussion

The ability to determine important dataset features based on the values of each optimizer parameters vital to improve the time consumption and accuracy of image classification. In addition, data augmentation techniques can increase the potential, of image classification. The parameters used to fine-tune the performance of each optimizer method [54] are shown in Table 7.

For training CNN models, MobileNet is a small model that requires little time for image classification; the highest accuracy of this experiment was 93.28%. This model is suitable for mobile computing device that require low power consumption for processing [45,46]. ResNet-50 is a popular classification model for predicting images; the highest accuracy in this experiment was 97.59%, which involved the problem solving of degradation by using identity mapping and residual mapping [26,27]. In this experiment, the best result of classification was 98.88%; this was accomplished by using DenseNet-121, which makes using of a bottleneck layer along with a construction for transition combinations and, using DenseBlock, offers a factor that indicates compression not exceeding a value of 1 [28,29]. There are limitations to this research. For example, our computer hardware features lower performance than the recommended requirements; thus, the application software for this experiment could not be used. Modern computer hardware has extremely high performance and can be used for large-scale image analysis.

Table 3, Table 4 and Table 5 compare the performance of the classification models using different optimization methods. The most accurate of the three CNN models was DenseNet-121 combined with Nadam and Mish, which provided an accuracy of 98.88%; the second-highest accuracy was ResNet-50 combined with Nadam and Mish, which provided an accuracy of 97.59%; and the third-highest accuracy was MobileNet combined with Nadam and Mish, which provided an accuracy of 93.28%. For a comparison of the time consumption of the classification models, the lowest time was found for MobileNet combined with RMSprop and ReLU, which provided a time consumption of 55 min 47 sec; the second-lowest time was ResNet-50 combined with RMSprop and ReLU, which provided a time consumption of 111 min 4 sec; and the third-lowest time was found for DenseNet-121 combined with RMSprop and ReLU, which provided a time consumption of 118 min 27 sec.

In order to speed up the training of the network, in this research we determined the batch size parameter based on the number of parameters used by each CNN model and the floating-point number of the activation function. Batch Size is the number of samples selected for a training session. A larger batch size will increase the learning speed of the model. Batch size directly affects the use of GPU memory. If the available GPU memory is not large, it is better to set the value smaller [55].

**Figure 12:**
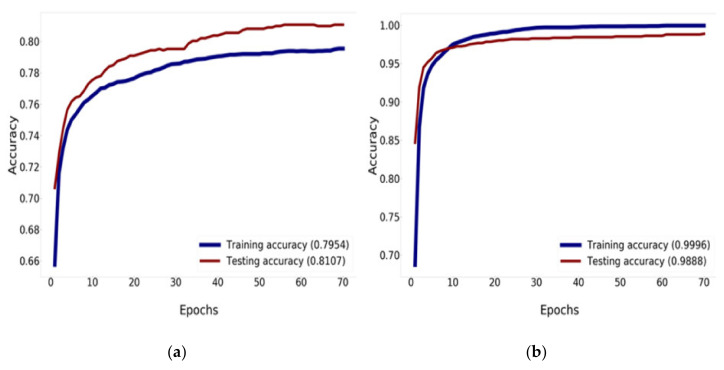
Training and validation accuracy history: (**a**) training and validation accuracy of DenseNet-121; (**b**) training and validation accuracy of DenseNet-121 combined with Mish and Nadam.

**Figure 13:**
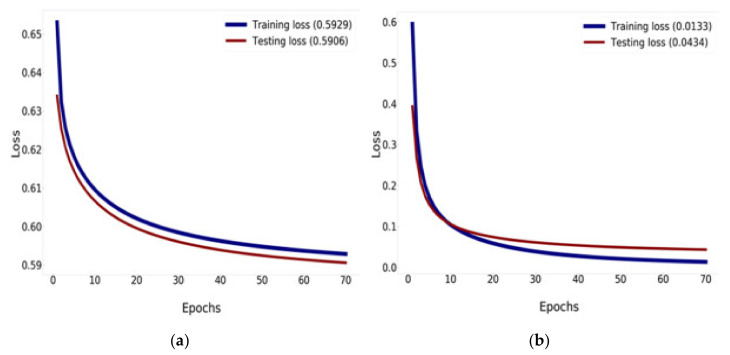
Training and validation loss history: (**a**) training and validation loss of DenseNet-121; (**b**) training and validation loss of DenseNet-121 combined with Mish and Nadam.

The Mish activation function is a new deep learning activation function that has a final accuracy better than Swish (+0.494%) and ReLU (+1.671%). In this work, Mish was superior to ReLU at high significance levels (*p* < 0.0001). The Mish function also guarantees the smoothness of each point. Mish has no upper bound but does, have a lower bound. Moreover, its smooth and non-monotonic properties all improve the results [34]. Figure 12 and Figure 13 illustrate tests of the validation accuracy.

Figure 12 compares the result of the training and validation accuracy between the traditional DenseNet-121 and DenseNet-121 combined with Mish and Nadam, which can increase the efficiency of accuracy up to 99.96% for training and 98.88% for validation, as shown in Figure 12b. DenseNet-121 can increase the efficiency of accuracy up to 79.54% for training and 81.07% for validation, as shown in Figure 12a. This research determined that 70 epochs are needed for training and validation history [56,57].

Figure 13 compares the results of the training and validation loss between the traditional DenseNet-121 and DenseNet-121 combined with Mish and Nadam, which can reduce the loss down to 0.0133% for training and down to 0.0434% for validation, as shown in Figure 13b. DenseNet-121 can reduce loss down to 0.5929% for training and 0.5906% for validation, as shown in Figure 13a.

**Figure 14:**
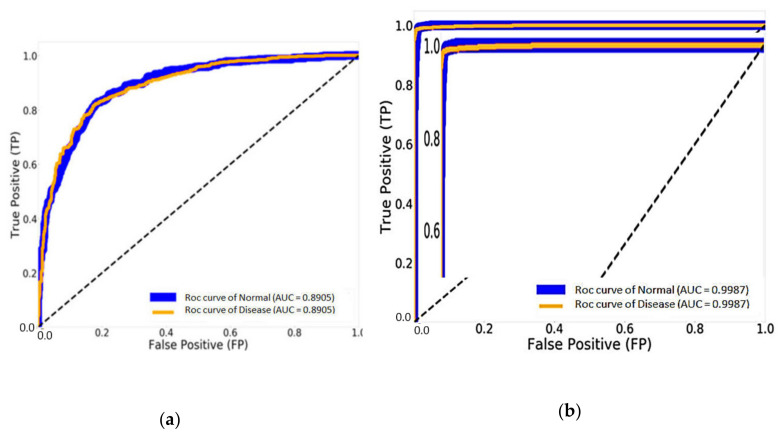
The results of the area under the curve (AUC) and receiver operating characteristics (ROC) curve: (**a**) DenseNet-121 and (**b**) DenseNet-121 combined with Mish and Nadam.

Figure 14 shows the result of the AUC and ROC curves produced by the FP and TP rates, which evaluate the performance of our CNN models. Figure 14a illustrates 89.05% of the AUC with the traditional DenseNet-121 and 99.87% of the AUC with DenseNet-121 combined with Mish and Nadam, as shown in Figure 14b. This experiment can improve the efficiency of traditional CNN models by changing hyperparameters using Mish and seven optimizer methods while adjusting the suitable values for each optimizer parameter to determine the best result.

## 6. Conclusions

This research was focused on applying modeling to detect traces of lung infection via a deep learning approach using the DenseNet-121 network, which was compared to other network models, such as ResNet-50 and MobileNet. The purpose of this research was to determine the efficiency of the three most well-known CNN models, MobileNet, Resnet-50 and Densenet-121, and to improve the efficiency of these CNN models by using Mish with seven optimizer methods to predict lung disease, as well as to compare the efficiency between traditional CNN models and CNN models using Mish with seven optimizer methods to predict lung disease.

The materials and methodology of this research was divided into four parts; the first part involved preparing the data method, which consisted of data augmentation techniques. Chest X-ray images featuring scoliosis of the spine in patients with abnormal lung shapes may look like lung disease symptoms and result in an erroneous diagnosis. Using the rotation technique in the data preprocessing stage for lung shape images can resolve this problem. Some areas of the chest X-ray image of a lung disease may look like a normal lung if the image processing technique is not suitable, which will lead to an incorrect diagnosis. Therefore, the suitable selection of an image processing technique to correctly classify lung disease is paramount. Using a preprocessing data technique can help create dataset training for the CNN model and increase efficiency. Related research has used a number of datasets with 5810 images for dataset training; this increased the processing time needed to create the CNN model.

The second part involved an activation function (Mish), transfer learning and dropout techniques to improve the efficiency of CNN models. Dropout was used to solve the overfitting problem and transfer learning was used to improve the efficacy of time consumption and the accuracy of image classification. The third part used a loss function (Cross-entropy) and seven optimizer methods consisting of Nadam, Adamax, Adam, Adadelta, RMSprop, Adagrad and SGD to determine the best CNN model performance to predict lung disease. The fourth part involved evaluating CNN models performance for lung disease prediction from the chest X-ray film.

Optimization is useful for model training; and involves the batch size, activation function and optimizer. Optimization is used to adjust the weight of the connected lines in a neural network. These seven optimizer methods can determine if the weight parameter needs to adjust the learning rate of the CNN model. Research on activation functions remains on going, and ReLU still dominates the activation functions used for deep learning; however, this research was changed by the introduction of Mish. This activation function determines the scale of the output variable value from the input variable value and also guarantees the smoothness of each point. Mish can receive a single scalar to change the network parameters without entering any scalar. Mish takes inspiration from Swish’s self-gating property, where scalar input is provided to the gate. Self-gating is able to replace activation functions like ReLU without changing the network parameters. Mish has no upper bound but does have a lower bound; further, its smooth and non-monotonic properties all improve the results [49]. Weights emphasize the importance of the input variable value that is used to determine the weight value of the input variable with the connected neuron before transferring the input variable value to the activation function. Weights can be changed by model training for the most accurate model [58,59].

There are limitations to this research. For example, our computer hardware features lower performance than the recommended requirements; thus, the application software for this experiment could not be used. Modern computer hardware has extremely high performance and can be used for large-scale image analysis. The creation of an efficient CNN model based on a number of images and preprocessing data techniques significantly improved the model’s efficiency. Some CNN structures are suitable parameters for dataset training to reduce time of training the CNN models and increase accuracy. Creating a CNN model using the rotation technique allowed us to customize rotation of the images by *−*10 to 10 degrees and train the dataset created by the CNN models [47,48].

Table 6 summarizes the performance of the model testing for the detection of lung lesions with a validation accuracy rate of 97.25%, for model testing performed using the traditional DenseNet-121 model. The validation accuracy rate was 98.88% and 98.97% for the model testing performed using DenseNet-121 combined with Mish and Nadam; this model gives the best performance for the detection of lung lesions and is better than the traditional DenseNet-121 model. Our research results improved the optimization of CNN models in the areas of each optimizer parameter, such as learning rate and activation function, which improved the performance efficiency of the CNN model to predict the lung disease from chest X-ray images. The results for time consumption under the different optimization methods showed the lowest time for MobileNet with RMSprop, at 55 min 47 sec; the second-lowest time was accomplished by MobileNet with Adadelta, at 56 min 20 sec, and the third-lowest time was found for MobileNet with Adagrad, at 56 min 36 sec. The contributions of using CNN models to predict lung lesions from chest X-ray images include assisting the doctor in reducing diagnostic time for detection and minimizing the errors in detecting lung lesions from chest X-ray images by choosing a suitable CNN structure for the chest X-ray dataset. With many numerous chest X-ray images, CNN models can be used to recognize image features. lung disease images can also be distorted by a scoliosis spinal condition.

For future studies, researchers should use deep learning to classify more sophisticated images. There are three patterns that can help develop this direction: the education of art and culture through the classification of artifacts; the development of agricultural business and economics through an evaluation of soil quality for planting economic crops with an analysis of plant leaf diseases; assisting in medical 3D organ simulation; and in the agricultural and food industries through the detection of cancer cells in humans.

## Figures and Tables

**Table 1:** The experimental environment.

Computer Equipment	Application and System Software
Central Processing Unit: Intel I7-6700 ^1^, 3.40 gigahertz	Operating System: Windows 10 ^2^
Random Access Memory: 32 gigabytes	Data Scikit-learn 0.21.2
Graphics Processing Unit (GPU): NVIDIA® GTX 1060 ^3^, 6 gigabytes	TensorFlow-GPU 1.13.1
Solid State Drive: 250 gigabytes	Python 3.7.3 and Keras-GPU 2.2.4

^1^ Central Processing Unit Intel I7-6700 is product name of Intel Corporation, Santa Clara County, CA, USA; ^2^ Windows 10 is product name of Microsoft Corporation, Washington, DC, USA; ^3^ NVIDIA® GTX 1060 is product name of NVIDIA Corporation, Santa Clara County, CA, USA.

**Table 2:** The learning rate of each optimizer method for training the CNN models.

Optimization Algorithm	SGD	Adagrad	Adadelta	RMSprop	Adam	Adamax	Nadam
Learning rate	0.01	0.01	0.01	0.001	0.001	0.002	0.002

Stochastic gradient descent (SGD).

**Table 3:** The comparative performance of MobileNet with Mish and ReLU using different optimizer methods.

Activation Function and Optimizer Methods	Accuracy %	Precision %	Recall %	F1 Measure %	Training Time
Nadam & ReLU	90.51	90.5	90.53	90.5	58 min 31 sec
Nadam & Mish	93.28	93.24	93.46	93.27	58 min 48 sec
Adamax & ReLU	87.17	87.31	86.9	87.04	58 min 05 sec
Adamax & Mish	90.44	90.42	90.64	90.43	58 min 18 sec
Adam & ReLU	86.91	87.19	86.56	86.75	58 min 27 sec
Adam & Mish	89.93	89.93	90.15	89.91	58 min 38 sec
Adadelta & ReLU	83.56	84.11	83.03	83.26	56 min 20 sec
Adadelta & Mish	87.6	87.54	87.73	87.57	56 min 34 sec
RMSprop & ReLU	83.04	83.68	82.47	82.71	55 min 47 sec
RMSprop & Mish	85.37	85.33	85.52	85.34	56 min 07 sec
Adagrad & ReLU	79.94	81.4	79.06	79.29	56 min 36 sec
Adagrad & Mish	80.89	80.8	80.94	80.84	57 min 03 sec
SGD & ReLU	74.48	75.93	73.48	73.52	59 min 27 sec
SGD & Mish	76.59	76.68	76.82	76.57	59 min 48 sec

**Table 4:** The comparative performance of ResNet-50 with Mish and ReLU using different optimizer methods.

Activation Function& Optimizer Methods	Accuracy %	Precision %	Recall %	F1 Measure %	Training Time
Nadam & ReLU	96.64	96.58	96.81	96.63	116 min 50 sec
Nadam & Mish	97.59	97.52	97.74	97.58	117 min 12 sec
Adamax & ReLU	94.23	94.2	94.43	94.22	111 min 21 sec
Adamax & Mish	95.09	95.07	95.31	95.08	111 min 42 sec
Adam & ReLU	93.45	93.42	93.65	93.44	113 min 39 sec
Adam & Mish	94.49	94.44	94.67	94.48	113 min 54 sec
Adadelta & ReLU	92.68	92.66	92.89	92.67	109 min 34 sec
Adadelta & Mish	93.54	93.51	93.74	93.53	109 min 39 sec
Rmsprop & ReLU	91.99	91.96	92.18	91.98	108 min 16 sec
Rmsprop & Mish	92.42	92.38	92.61	92.41	109 min 12 sec
Adagrad & ReLU	84.76	84.71	84.62	84.66	111 min 04 sec
Adagrad & Mish	87.17	87.09	87.12	87.11	111 min 34 sec
SGD & ReLU	79.43	79.34	79.24	79.28	117 min 42 sec
SGD & Mish	79.69	79.6	79.5	79.54	118 min 12 sec

**Table 5:** The comparative performance of DenseNet-121 with Mish and ReLU using different optimizer methods.

Activation Function& Optimizer Methods	Accuracy %	Precision %	Recall %	F1 Measure %	Training Time
Nadam & ReLU	98.62	98.56	98.69	98.61	123 min 10 sec
Nadam & Mish	98.88	98.83	98.91	98.87	123 min 29 sec
Adamax & ReLU	95.69	95.65	95.9	95.68	118 min 27 sec
Adamax & Mish	96.38	96.32	96.54	96.37	118 min 39 sec
Adam & ReLU	95.09	95.02	95.22	95.08	119 min 40 sec
Adam & Mish	95.43	95.37	95.59	95.42	120 min 17 sec
Adadelta & ReLU	91.82	91.75	91.83	91.78	116 min 22 sec
Adadelta & Mish	92.34	92.27	92.34	92.3	116 min 47 sec
RMSprop & ReLU	89.15	89.12	89.05	89.08	114 min 29 sec
RMSprop & Mish	91.65	91.59	91.62	91.6	115 min 09 sec
Adagrad & ReLU	85.19	85.29	84.92	85.04	117 min 48 sec
Adagrad & Mish	88.03	88.07	87.85	87.93	118 min 05 sec
SGD & ReLU	81.06	81.12	80.74	80.86	123 min 45 sec
SGD & Mish	82.53	82.69	82.15	82.31	123 min 57 sec

**Table 6:** Comparative performance of model testing using DenseNet-121 combined with Mish and Nadam and the traditional method of DenseNet-121.

Models	Optimizers	Classes	Images	True Prediction	False Prediction
DenseNet-121 (Traditional method)	SGD	Normal	232 (100%)	224 (96.56%)	8 (3.44%)
		Disease	348 (100%)	340 (97.70%)	8 (2.30%)
		Overall	580 (100%)	564 (97.25%)	16 (2.75%)
DenseNet-121 and Mish	Nadam	Normal	232 (100%)	229 (98.71%)	3 (1.29%)
		Disease	348 (100%)	345 (99.13%)	3 (0.87%)
		Overall	580 (100%)	574 (98.97%)	6 (1.03%)

**Table 7:** Parameters of each optimizer method.

Optimizer Methods	Parameters
Nadam	Learning rate, beta_1, beta_2, epsilon, schedules-decay
Adamax	Learning rate, beta_1, beta_2, epsilon, decay
Adam	Learning rate, beta_1, beta_2, epsilon, decay
Adadelta	Learning rate, rho, epsilon, decay
RMSprop	Learning rate, rho, epsilon, decay
Adagrad	Learning rate, epsilon, decay
SGD	Learning rate, momentum, decay, nesterov
